# Characterization of the *Verticillium dahliae* Exoproteome Involves in Pathogenicity from Cotton-Containing Medium

**DOI:** 10.3389/fmicb.2016.01709

**Published:** 2016-10-28

**Authors:** Jie-Yin Chen, Hong-Li Xiao, Yue-Jing Gui, Dan-Dan Zhang, Lei Li, Yu-Ming Bao, Xiao-Feng Dai

**Affiliations:** Laboratory of Cotton Disease, Institute of Food Science and Technology, Chinese Academy of Agricultural SciencesBeijing, China

**Keywords:** *Verticillium dahliae*, exoproteome, CAZymes, plant cell wall degradation enzyme, pectinases

## Abstract

*Verticillium* wilt, caused by the *Verticillium dahliae* phytopathogen, is a devastating disease affecting many economically important crops. Previous studies have shown that the exoproteome of *V. dahliae* plays a significant role in this pathogenic process, but the components and mechanisms that underlie this remain unclear. In this study, the exoproteome of *V. dahliae* was induced in a cotton-containing C’zapek-Dox (CCD) medium and quantified using the high-throughput isobaric tag technique for relative and absolute quantification (iTRAQ). Results showed that the abundance of 271 secreted proteins was affected by the CCD medium, of which 172 contain typical signal peptides generally produced by the Golgi/endoplasmic reticulum (ER). These enhanced abundance proteins were predominantly enriched in carbohydrate hydrolases; 126 were classified as carbohydrate-active (CAZymes) and almost all were significantly up-regulated in the CCD medium. Results showed that CAZymes proteins 30 and 22 participate in pectin and cellulose degradation pathways, corresponding with the transcription levels of several genes encoded plant cell wall degradation enzyme activated significantly during cotton infection. In addition, targeted deletion of two pectin lyase genes (*VdPL3.1* and *VdPL3.3*) impaired wilt virulence to cotton. This study demonstrates that the *V. dahliae* exoproteome plays a crucial role in the development of symptoms of wilting and necrosis, predominantly via the pathogenic mechanisms of plant cell wall degradation as part of host plant infection.

## Introduction

*Verticillium* wilt, caused by the phytopathogen *Verticillium dahliae*, is an extremely destructive disease affecting hundreds of dicotyledonous plant species. This disease is particularly hard to eradicate and manage because the survival structures produced by the wilt pathogen remain viable in soil for more than two decades, persistently threatening crops (Fradin and Thomma, 2006; Klosterman et al., 2009). Similar to other known pathogens of this type, *V. dahliae* colonizes and proliferates in the vascular (xylem) system of a plant, disrupting water-conduction, and leading to a series of characteristic symptoms including wilting leaves, stunting, necrosis, and vein clearing (Klosterman et al., 2009). Typically, *V. dahliae* enters the host plant either via the root or at the sites of lateral root formation (Bishop and Cooper, 1983), before traversing the endodermis, which normally acts as a physical barrier to pathogen infection (Schnathorst, 1981; Vallad and Subbarao, 2008). When the pathogen reaches the vascular tissue, hyphae start to bud and form conidia which are carried by the xylem fluid and trapped at vessel end walls before germinating and penetrating adjacent vessel elements to continue colonization and start another infection cycle (Cooper and Wood, 1975; Bishop and Cooper, 1983). The pathogen produces toxic or elicitor-like substances as well as hydrolytic enzymes that facilitate its colonization and proliferation in the unique plant vascular system environment (Fradin and Thomma, 2006; Klosterman et al., 2009). Given this background, the *V. dahliae* exoproteome, including secreted proteins, is thought to play an important role in determining pathogenicity (Fradin and Thomma, 2006).

The arsenal of proteins secreted by plant pathogens have been the focus of recent studies as these modulate interactions between microbes and their hosts. The pathogen exoproteome, comprising multiple pathogenic factors, is likely to be involved in the infection process (Rep, 2005; Kamoun, 2006; Bent and Mackey, 2007; Thomma et al., 2011). Examples include LysM effectors from the tomato leaf mold fungus *Cladosporum fulvum* and wheat leaf blotch fungus *Mycosphaerella graminicola* (de Jonge et al., 2010; Marshall et al., 2011), necrosis and ethylene-inducing-like protein (NLP) genes from the *Verticillium* wilt fungus *V. dahliae* (Zhou et al., 2012; Santhanam et al., 2013), and small cysteine-rich proteins (SCRPs) from the vascular wilt fungus *Fusarium oxysporum* f. sp. *Lycopersici* and the rice blast fungus *Magnaporthe oryzae* (Lievens et al., 2009; Valent and Khang, 2010). A number of studies have focused on the exoproteome using proteomic and genomic techniques (Spanu, 2012; Girard et al., 2013) as this is thought to play an important role in pathogenesis. Investigations include studies on *Fusarium* spp. (Phalip et al., 2005; Paper et al., 2007; Ravalason et al., 2012), *Magnaporthe oryzae* (Wang et al., 2011; Jung et al., 2012), *Xanthomonas oryzae* (Wang et al., 2013), and *Botrytis cinerea* (González-Fernández et al., 2014). A deletion mutant of the *Verticillium* transcription regulator *Vta2*, which controls expression of 125 secreted proteins, failed to colonize plants and induce disease symptoms, indicating a role in pathogenicity (Tran et al., 2014), and the genome of two strains of the vascular wilt fungus *V. dahliae*, VdLs.17 and JR2, isolated from lettuce and tomato, respectively, have been sequenced (Klosterman et al., 2011; de Jonge et al., 2013). Comparative genomic analyses have also shown that *V. dahliae* has markedly increased secretion levels of pathogenic factors, including LysM effectors, NLPs, and an Ave1 virulence factor (Klosterman et al., 2011; de Jonge et al., 2012, 2013; Zhou et al., 2012; Santhanam et al., 2013; Klimes et al., 2015), in agreement with previous reports that the purified exoproteome of this pathogen can induce chlorosis and necrosis on the leaves of susceptible plants (Buchner et al., 1982, 1989; Nachmias et al., 1985; Meyer et al., 1994; Mansoori et al., 1995; Davis et al., 1998). These results all suggest that *V. dahliae* requires the presence of exoproteome virulence factors in order to infect host plants.

Hydrolases in the exoproteome are considered important for the generation of disease symptoms and pathogenesis, especially the enzymes involved in plant cell wall degradation (King et al., 2011; Glass et al., 2013; Kubicek et al., 2014). Indeed, genomic analysis has demonstrated that the expansion of PCWDEs is closely related to pathogenicity. This is especially true in the case of pectinases and cellulases, which participate in plant cell wall (pectin and cellulose) degradation (Lévesque et al., 2010; Ma et al., 2010; Ospina-Giraldo et al., 2010; Amselem et al., 2011; Klosterman et al., 2011). In *V. dahliae*, the pathogenic role of several PCWDEs has been identified; disruption of sucrose non-fermenting 1 (*VdSNF1*), which regulates catabolic repression, resulted in the reduced expression of a large number of PCWDE genes and in severely impaired virulence to plants (Tzima et al., 2011). Similarly, a knockout mutant in the glucosyltransferase homolog gene VDAG_02071 resulted in drastically reduced disease symptoms in *Nicotiana benthamiana* compared with the wild-type strain (Klosterman et al., 2011), while deletion of the *VdSSP1* gene, the product of which is a secretory protein involved in pectin and starch degradation, reduced virulence levels on cotton (Liu Y. et al., 2013). These results therefore suggest that *V. dahliae* utilizes several CWDEs to infect host plants. Previous research has also shown that pectinolytic enzymes in the exoproteome of *Verticillium* spp. play a critical role in pathogenesis; these include polygalacturonase, pectate lyase, and pectinesterase, which induce necrosis of plant tissues *in vitro* and cause wilt symptoms (Wang and Keen, 1970; Mussel and Strause, 1972; Cooper and Wood, 1980; Huang and Mahoney, 1999). A comparative genomic analysis showed that *Verticillium* spp. increased pectinolytic enzyme activities to accelerate plant cell wall degradation, especially polysaccharide lyase (PL) and glycoside hydrolase (GH) gene families (Klosterman et al., 2011). Thus, PCWDEs in the exoproteome of *V. dahliae*, especially pectinases and cellulases, likely play a crucial role in pathogenesis.

The iTRAQ method, coupled with liquid chromatography-tandem mass spectrometry (LC–MS/MS), has previously been utilized in proteomics analysis. The iTRAQ technique facilitates the quantification of protein concentrations in biological systems and has been applied, for example, to *Trichoderma reesei* (Adav et al., 2013), *Phanerochaete chrysosporium* (Adav et al., 2012), *Aspergillus fumigatus* (Liu D. et al., 2013), and *Arthrobotrys oligospora* (Liang et al., 2013). However, although a number of *Verticillium* spp. proteomes have been reported (Mandelc et al., 2009, 2013; El-Bebany et al., 2010; Mandelc and Javornik, 2015), no data are available on the exoproteome associated with the interactions between *V. dahliae* and host plants. Thus, in this study, the nutritional components of the unique *in vitro* CCD medium were used to evaluate the induced secretome of *V. dahliae*. The pathogenic function of the induced secretome was analyzed, and its components were identified. The pathogenic function of proteins was analyzed by knocking out their encoding genes. This study provides insights into the mechanism by which the proteins secreted by *V. dahliae* facilitate its adaption to the unique environment of the plant vascular system.

## Materials and Methods

### Strain Cultivation and Exoproteome Extraction

The high virulence defoliating strain of *V. dahliae*, Vd991, was used for exoproteome analysis. This strain was grown on a potato dextrose agar (PDA) medium for 7 days to enable the production of conidia, before these were harvested by washing the plate with sterilized water. The resulting suspension was then filtered through gauze to remove mycelia and conidia concentration was adjusted to 5 × 10^6^ conidia/mL. The CCD medium was prepared by sowing a susceptible cultivar of *Gossypium hirsutum* L., ‘Junmian No.1’, in sterilized soil at 28°C under a photoperiod of 14 h light/10 h dark for 3 weeks. Subsequently, whole plants without leaves were harvested and ground into powder in liquid nitrogen, and the CCD medium was prepared using a C’zapek-Dox (CD, i.e., 2 g/L NaNO_3_, 0.5 g/L MgSO_4_-7H_2_O, 0.5 g/L KCl, 100 mg/L 14 FeSO_4_-7H_2_O, and 1 g/L K_2_HPO_4_, pH 7.2) solution supplemented with 1% (w/v) cotton tissue powder.

In order to induce the exoproteome, 5 mL (5 × 10^6^ conidia/mL) of prepared Vd991 conidia were cultured on the CCD medium and CD medium. Three replicates of this preparation were incubated at 25°C and centrifuged at 180 rpm. Under normal conditions, conidia were incubated in the original CD medium supplemented with 1% (w/v) sucrose but without the 1% (w/v) cotton tissue powder; at the same time, two media were cultured in the same conditions but without inoculating Vd991 conidia to create a control group. After 5 days of incubation, culture suspensions were filtered through four layers of gauze and the fungal biomass was further removed via centrifugation. Supernatants were collected and the solution was filtered through a 0.22 μm membrane (Millipore, Temecula, CA, USA). The Avant purification system (GE Healthcare, Piscataway, NJ, USA) was used for exoproteome enrichment; the filtered solution was enriched using a Hitrap TM Capto SP/Q column (GE Healthcare, New Jersey, USA), desalted using sephasex G75 (GE Healthcare, Piscataway, NJ, USA). Finally, the purified exoproteome was freeze-dried before being resolved in sterilized water. Exoproteome concentration was then determined using 2-D Quant kit assay reagents (GE Healthcare, Piscataway, NJ, USA) following the manufacturer’s instructions.

### Wilting Activity Determination

A small amount (1.0 μg) of purified exoproteome protein was dissolved in 20.0 μL sterilized water in a 0.5 mL microcentrifuge tube in order to determine wilting activity. To do this, *G. hirsutum* L., ‘Junmian No.1’ cotyledons containing petiole were collected from 2-week-old seedlings and were inserted directly into the microcentrifuge tube containing the exoproteome solution. When all this solution was absorbed by the cotyledons, 2 mL of sterilized water was added to the tube and it was placed in a box with a transparent cover to maintain appropriate moisture. Cotyledon phenotypes were investigated after 72 h at 25°C using an illumination incubator. Four treatments were carried out for the wilting activity assay, including the induced exoproteomes purified from the CCD medium, the CD medium, and the blank control of the two types. In each treatment, 15 cotton cotyledons were detected.

### iTRAQ Labeling, Spectrometric Analysis, and Data Processing

Samples containing 100 μg of protein were digested using Trypsin Gold (Promega, Fitchburg, WI, USA) for 16 h at 37°C to generate peptides. Subsequent to digestion, these peptides were dried by vacuum centrifugation, and resuspended in 0.5 M TEAB. Desalted peptides were then labeled with iTRAQ reagents (Applied Biosystems, Carlsbad, CA, USA) following the manufacturer’s instructions (Zieske, 2006). Thus, three repeats of extracted proteins in the CD medium were labeled with reagents 113, 114, and 115, while the fractions in the CCD medium were labeled with reagents 116, 117, and 118, respectively. Three technical repeats of each treatment were performed, and the peptide mixtures were pooled and dried by vacuum centrifugation. These pooled iTRAQ-labeled peptide mixtures were fractionated into ten portions using strong cationic exchange chromatography and desalted in a nanobored C18 column with a picofrit nanospray tip (i.e., 365 μm i.d × 15 cm, 5 μm particles, 150 À pore size) (Phenomenex Inc., Torrance, CA, USA).

Data acquisition was performed using time-of-flight (TOF)-MS in a TripleTOF5600 MS system (AB SCIEX, Concord, ON, Canada) fitted with a Nanospray III source (AB SCIEX) and using a pulled quartz tip as the emitter (New Objectives, Woburn, MA, USA). Data were acquired using an ion spray voltage of 2.5 kV, a curtain gas of 30 PSI, a nebulizer gas of 15 PSI, and an interface heater temperature of 150°C. The MS was operated with a resolving power of greater than or equal to 30,000 full width at maximum (FWHM) for TOF scans. Instrument data files (.wiff) for fractions were transformed to.mgf files using MS convertion (AB SCIEX, Concord, ON, Canada) before being merged into one.mgf.

Raw files were processed using the Mascot computational proteomics platform version 2.3.01. Fragmentation spectra were searched against the Vd991 genome encoding protein database (9,818 genes) maintained in our laboratory with fragment mass tolerances set to 20 mmu and with up to one missed cleavage. For database searches, carbamidomethylation of cysteine was set as a fixed modification, while the oxidation of glutamine and protein N-terminal acetylation were chosen as variable modifications. Both peptide and protein identifications were filtered at 1% false discovery rates so proteins identified with at least two unique peptides were independent of peptide score.

### Exoproteome Annotation

Secretory proteins were annotated on the basis of subcellular localization, performed using the WoLF PSORT software (fungi model) to identify putative extracellular proteins (Horton et al., 2007). Signal peptides and signal peptide cleavage sites of putative extracellular proteins were predicted using the SignalP software (version 4.1; D-Score cut-off set to 0.500) (Petersen et al., 2011). All putative extracellular proteins with signal peptides were then analyzed for the presence of transmembrane domains using the TMHMM 2.0 software (Krogh et al., 2001), while those containing just a signal peptide but lacking transmembrane domains, as predicted by either of the two software packages, were identified as secreted proteins. Non-classical secretory proteins (NCSPs) were predicted using the SecretomeP software (Bendtsen et al., 2004), and annotation of putative CAZymes was performed using the Hidden Markov Model (HMM)-based routine of the CAZymes database (Cantarel et al., 2009). Significant hits compared to this database were identified using the set of putative CAZymes in the BLAST software (i.e., *e*-value < 1e^-5^, similarity >30%) (Altschul et al., 1997), subsequently used to increase accuracy of annotation. The CAZymes involved in plant cell wall degradation were then classified using methods outlined in previous studies (Battaglia et al., 2011; Goodwin et al., 2011), while the homologues of known pathogenicity-related genes were predicted using the Pathogen-Host Interactions (PHI) database (version 3.6)^1^ (Winnenburg et al., 2008). Proteins comprised of less than 400 amino acids and containing up to four cysteine residues were designated as SCRPs, and pathways were annotated using the Putative functional annotations were interrogated to known databases using BLASTP to identify the best homologues, including the eggNOGs (Powell et al., 2012), InterProScan (Jones et al., 2014), and Gene Ontology (GO). The significance of GO catalog for induced exoproteome was identified using a *Fisher’s* Exact Test (filtered with *FDR* ≤ 0.05). The Web Gene Ontology Annotation Plot (WEGO) software was used to retrieve GO annotations of unigenes for describing biological processes, molecular functions and cellular components (Ye et al., 2006). Kyoto Encyclopedia of Genes and Genomes (KEGG) database implemented in the BlastP software (Kanehisa et al., 2012).

### Agrobacterium-Mediated Targeted Gene Deletion

In order to generate the deletion constructs of *PL3* genes, flanking sequences were obtained from Vd991 genomic DNA. Thus, the hygromycin element, which consists of the *TrpC-*promoter, the hygromycin phosphotransferase gene, and the *Nos-*terminator, was amplified in our laboratory with Hyg-F and Hyg-R primers from the pCT-Hyg vector. These three fragments were fused into one via fusion PCR while nest PCR was carried out to obtain the amplicon, which included the upstream fragment, hygromycin elements, and the downstream fragment. Amplified products were then cloned into a pGKO2-Gateway vector (Khang et al., 2005) as previously described (Liu Y. et al., 2013). All the primers used for vector construction are listed in Supplementary Table S7; the recombinant vectors for the deletion mutant were transferred into *Agrobacterium tumefaciens* AGL-1 for fungal transformation.

The *A. tumefaciens*-mediated transformation of *V. dahliae* was carried out for the homolog recombination and complementary transformants as previously described (Mullins and Kang, 2001). Homologous recombination transformants were selected on the PDA medium (i.e., potato, 200 g/L, glucose, 20 g/L, agar, 15 g/L) with 200 μg/mL of cefotaxime, 50 μg/mL of hygromycin, and 200 μg/mL of 5-fluoro-2′-deoxyuridine. Single spore isolation was performed for all transformants with positive ones verified using PCR.

### Pathogenicity Assays

Pathogenicity assays were performed on cotton seedlings when the first euphylla was developed; 5 × 10^6^ conidia/mL suspensions in sterile water were prepared, and six pots of cotton seedlings with three repeats were prepared for each transformant. Each pot containing five cotton seedlings was fully immersed in a new tray previously immersed in a 15 mL conidial suspension. Seedlings were then grown and maintained at 28°C under a 14 h light/10 h dark photoperiod, and disease symptom phenotype was investigated 3 weeks after inoculation.

### Gene Expression Analysis

To determine the *in planta* expression of *PL3* genes, cotton seedlings were root inoculated with 5 × 10^6^ conidia/mL of a *V. dahliae* suspension. Whole plants were harvested at 3, 5, 7, 9, and 14 days post inoculation (dpi) and flash frozen in liquid nitrogen for RNA extraction using a Total RNA Miniprep Kit (Axygen, Tewksbury, MA, USA) and cDNA synthesis using a FastQuant cDNA Reverse Transcriptase Kit (TianGen, Beijing, China). Quantitative reverse transcription-PCR (qRT-PCR) was performed to identify expression levels of PCWDE genes affected by T-DNA insertion using the FastFire qPCR premix, SYBR Green (TianGen, Beijing, China), and relative gene expression levels were calculated using the 2^-ΔΔCT^ method (Livak and Schmittgen, 2001) with the β-tubulin gene (VDAG_10074, VdLs.17) as an internal control. All primers are listed in Supplementary Table S3; real-time PCR conditions comprised an initial 94°C denaturation step for 10 min, followed by 40 cycles at 94°C for 15 s and at 60°C for 1 min.

## Results

### The Induced *V. dahliae* Exoproteome Displays Cytotoxic Activity on Cotton

**FIGURE 1:**
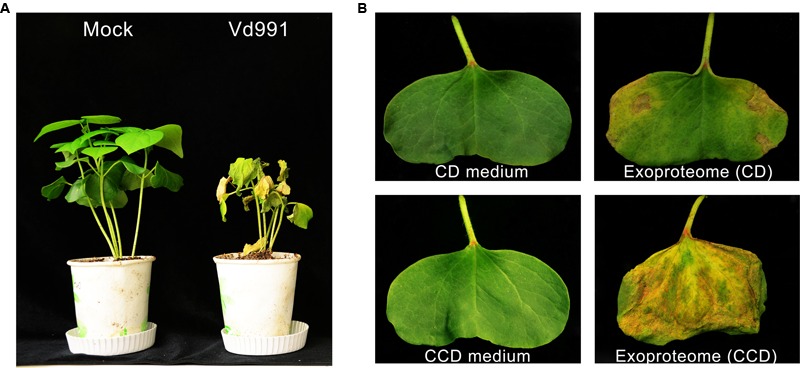
**Cytotoxic activity of the induced exoproteome of the *V. dahliae* strain Vd991. (A)** Virulence detection of Vd991 infection on cotton. Two-week-old seedlings of susceptible cotton of *G. hirsutum* L., ‘Junmian No.1’ were inoculated with sterile water (Mock), wild-type (WT) *V. dahliae*. The disease symptom phenotype was investigated 3 weeks after inoculation. **(B)** Validation of the cytotoxic activity of the Vd991 exoproteome on cotton leaves. CD medium, C’zapek-Dox medium; CCD medium, cotton-containing C’zapek-Dox medium. Wilting activity of an exoproteome purified from a culture suspension was evaluated on cotton cotyledons. The CD medium and CCD medium were used as the negative controls.

Strain Vd991 of *V. dahliae*, isolated from a *G. hirsutum* host, is a highly virulent strain that causes *Verticillium* wilt in cotton (**Figure 1A**). To evaluate the cytotoxic activity of these secreted proteins, wilting activity of an exoproteome purified from a culture suspension was evaluated on cotton cotyledons. Utilizing sucrose as the sole carbon source in the CD medium, the exoproteome of *V. dahliae* exhibited little cytotoxic activity on cotton leaves, which showed few wilting spots (**Figure 1B**). However, the induced exoproteome of *V. dahliae* purified from modified medium containing the CCD medium caused serious chlorosis and necrosis of cotton leaves (**Figure 1B**). These results indicate that the induced exoproteome possesses significant cytotoxic activity and causes symptoms similar to those induced by inoculation with *V. dahliae* strains.

### Identification of the Induced Exoproteome Components of *V. dahliae*

A total of 1,881 suspected proteins were identified using a concatenated target and decoy strategy with Vd991 genome sequences (9,818 protein-encoding sequences) (Supplementary Figure S1; Supplementary Table S1). Using cut-off values based on unique alignment peptides ≥2, *FDR* < 0.01, and 1.5-fold change, 536 proteins were quantified by iTRAQ using three biological replicates. Next, two-way ANOVA of fold changes (model: fold change = medium conditions + replicates + system error) in the three biological replicates and filtering of regulated proteins on the basis of a *P*-value < 0.00001 allowed us to identify 325 proteins with highly significant fold change abundances in the induced exoproteome compared to those from the original CD medium (Supplementary Figure S1; Supplementary Table S2). Of the 325 differentially expressed proteins, abundances of 230 were higher in the CCD medium compared with that which used sucrose as the carbon source (Supplementary Table S2). These results suggest that the *V. dahliae* pathogens up-regulate the expression of most exoproteome components to adapt to the cotton plant tissue environment.

### The Induced Exoproteome Is Predominantly Produced by the Golgi/Endoplasmic Reticulum (ER) Secretory System in *V. dahliae*

Although the exoproteome in this study was purified from culture filtrate, some intracellular proteins were likely present due to cytolysis during cultivation. This was confirmed by evolutionary genealogy of genes: Non-supervised Orthologous Groups (eggNOG) annotation of the induced exoproteome which shows intracellular localization of several proteins, 54 of which are involved in information storage and processing, including ribosomal proteins and eukaryotic translation initiation factors (**Table 1**; Supplementary Table S3). This result also indicates that a small number of intracellular proteins were present in the induced exoproteome due to normal cytolysis of *V. dahliae*.

**Table 1:** Secretory analysis of inducing exoproteome by bioinformatics.

Description	Number ofproteins in Vd991
Differentially expressed proteins quantified by iTRAQ-based	325
Proteins involve in information storage and processing	54
Proteins involve in the secretory	271
Proteins secreted by the Golgi/ER secretory systems	172
Proteins secreted by the unconventional secretory systems	15
Proteins secreted by the yet unknown secretory systems	84

The subcellular localization of identified proteins was predicted using multiple software tools. Results showed that the proteins predicted to be extracellular via WoLF-PSORT and to harbor a signal peptide by SignalP4.1, but lacking a transmembrane domain as predicted by TMHMM2.0, were secreted proteins generally from the Golgi/ER systems. With the exception of the 54 intracellular proteins discussed above, 172 of the 271 with affected abundances were identified as secreted (**Table 1**; Supplementary Figure S1; Supplementary Table S4). However, because some extracellular proteins do not contain a signal peptide, these were subjected to SecretomeP analysis to identify NCSPs. In this way, 15 proteins lacking a transmembrane domain were identified as NCSPs by filtering those with NN scores > 0.5 using the SecretomP software (**Table 1**; Supplementary Figure S1; Supplementary Table S4). In total, 69% (187/271) of predicted proteins in the induced exoproteome were identified as secreted (**Table 1**). The ratio of typical secreted proteins containing a signal peptide was significantly higher than NCSPs in the induced exoproteome (**Table 1**), indicating that secreted proteins of *V. dahliae* were produced mainly by the Golgi/ER system. In addition, 31% of the remaining identified proteins (84) were not predicted by bioinformatic analysis to be secretory (**Table 1**; Supplementary Figure S1), suggesting that the *V. dahliae* pathogen possesses as yet unknown secretory mechanisms.

### Function of the Induced Exoproteome in *V. dahliae* Pathogenicity

**FIGURE 2:**
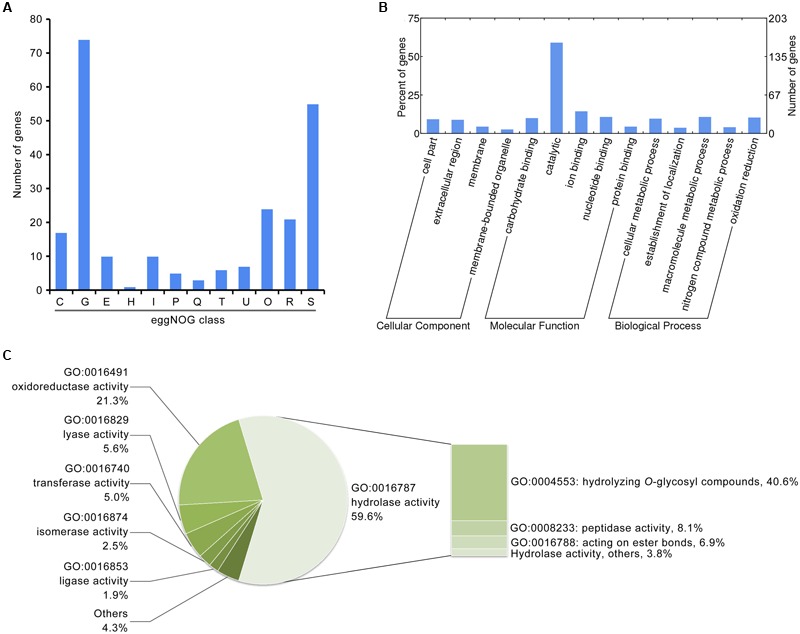
**Functional annotation of the *V. dahliae* induced exoproteome. (A)** Functional annotation of the induced exoproteome using the eggNOG database. The alphabet in *x*-axis represents the eggNOG class. T, signal transduction mechanisms; U, intracellular trafficking, secretion, and vesicular transport; O, posttranslational modification, protein turnover, chaperones; C, energy production and conversion; G, carbohydrate transport and metabolism; E, amino acid transport and metabolism; H, coenzyme transport and metabolism; I, lipid transport and metabolism; P, inorganic ion transport and metabolism; Q, secondary metabolites biosynthesis, transport and catabolism; R, general function prediction only; S, function unknown. **(B)** GO annotation of the *V. dahliae* induced exoproteome. The significance of GO annotations for induced exoproteome was identified using a *Fisher’s* Exact Test (*FDR* ≤ 0.05), and functional clusters was performed using WEGO software. **(C)** Function catalog of the induced exoproteome by catalytic class (GO:0003824).

Previous genomic studies on *Verticillium* spp. suggest a gene family expansion such that encoded secreted proteins are involved in plant cell wall degradation, a process which likely facilitates proliferation of the fungi in the unique environment of the plant vascular system (Klosterman et al., 2011). Thus, to identify factors related to pathogenicity in the induced exoproteome, the function of proteins present at enhanced abundance was analyzed via annotation using several databases. Results show that of the 271 proteins that have enhanced abundance in the induced exoproteome, at least 74 are predicted to be involved in carbohydrate transport and metabolism based on eggNOG annotation (**Figure 2A**). Indeed, GO annotation showed that 160 proteins occurring at enhanced abundance are involved in molecular catalytic function (**Figure 2B**), including 95 that are enriched in hydrolase activity, principally the hydrolysis of *O*-glycosyl compounds (**Figure 2C**). These results therefore suggest that the induced exoproteome plays a crucial role in *V. dahliae* carbohydrate metabolism in the CCD medium.

In addition, several enhanced abundance proteins in the induced exoproteome are biologically involved with reactive oxygen species, and 17 of these are clustered in energy production and conversion according to eggNOG annotation (**Figure 2B**). Overall, 34 proteins are oxidoreductase active, according to the GO annotation (**Figure 2C**). Partially secreted proteins are referred to as effectors, and 16 hypothetical proteins (i.e., without homologue function) belonging to the small cysteine-rich protein family (< 400 amino acids, ≥4 cysteine residues; Supplementary Table S5) were identified in the induced exoproteome. These probably function to facilitate pathogen colonization by modulating host biochemistry and physiology. In addition, the abundance of known effectors NLPs, were significantly up-regulated 9.05 ± 2.04 and 3.75 ± 0.17 fold in the CCD medium, respectively (Supplementary Table S5). These results suggest that the functions of these enhanced abundance proteins in the induced exoproteome are closely associated with the pathogenicity of *V. dahliae* to cotton.

### The Induced Exoproteome Is Significantly Associated with Plant Cell Wall Degradation

**FIGURE 3:**
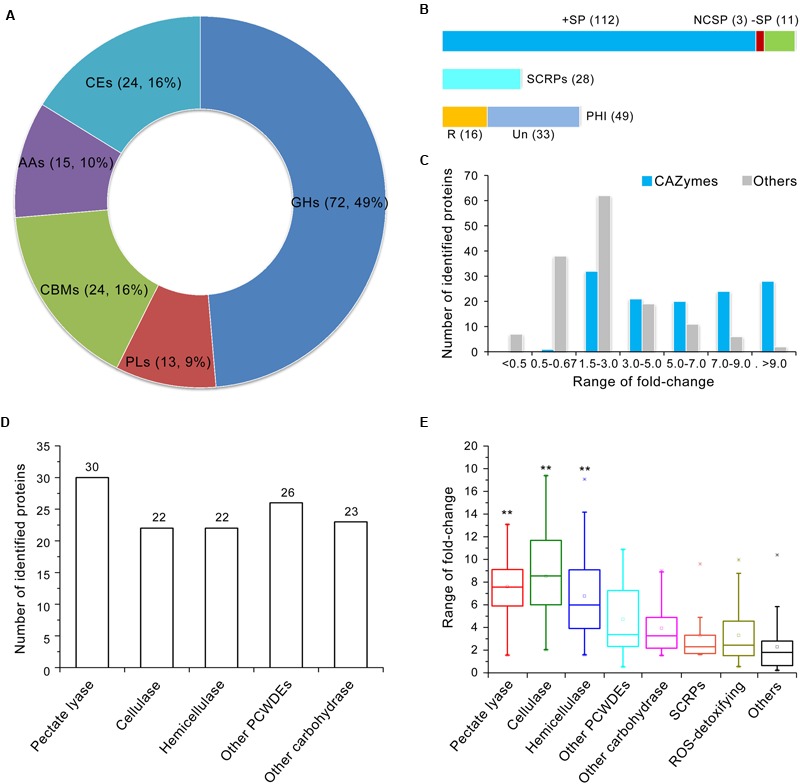
**Annotation of CAZymes in the *V. dahliae* induced exoproteome. (A)** CAZymes catalog of the induced exoproteome. GHs, glycoside hydrolases; PLs, polysaccharide lyases; CBMs, carbohydrate-binding modules; AAs, auxiliary activities; CEs, carbohydrate esterases. Two numbers in bracket represents the quantity and ratio of the total CAZymes. **(B)** Analysis of the potential pathogenic function of CAZymes. The upper bar is the secretory characteristic of CAZymes, the bar in blue, red and green color represent the CAZymes contains typical signal peptide, non-classical secretory proteins, and without signal peptide, respectively; the middle bar represents CAZymes belongs to SCRPs; the bottom bar represents CAZymes belongs to PHI protein, the bar in yellow and watchet represents the CAZymes homolog to PHI that reduced (R) virulence and unaffected (UN) pathogenicity, respectively. **(C)** Distribution of abundance variation of CAZymes in the induced exoproteome. The blue and gray bar represents the quantity of identified CAZymes and other identified proteins among different variation range in induced exoproteome, respectively. **(D)** Statistics showing the number of identified proteins involved in plant cell wall degradation in the induced exoproteome. **(E)** Abundance variations of identified proteins involved in cell wall degradation. The range of fold-change was displayed by the Box whisker plot. In each box plot, the central point represents the median, the rectangle gives the interval between the 25 and 75% percentiles, and the whisker indicates the range, the color asterisk (^∗^) represents the extreme outlier. Significant of abundance variations in pectate lyase, cellulase, and hemicellulose to other proteins were identified using a Student’s *t*-test with a significance threshold of 0.01(^∗∗^).

The induced exoproteome is enriched in hydrolase activity, principally *O*-glycosyl compounds (**Figure 2C**), which implies involvement in carbohydrate metabolism. To confirm this hypothesis, further functional annotation of the proteins identified in the induced exoproteome was performed using the CAZymes database (Yin et al., 2012). In total, 126 of the 271 enhanced abundance proteins were recognized as CAZymes, 105 of which contained only one functional domain (CAZymes module) (Supplementary Table S6). More than half of these proteins (*n* = 72) contained a GH module, which facilitates hydrolysis and/or rearrangement of glycosidic bonds. In addition, 24 proteins contained carbohydrate-binding modules (CBM), which promote the hydrolase activity of carbohydrate hydrolase (**Figure 3A**; Supplementary Table S6), while 13, 15, and 24 proteins exhibited PL, carbohydrate esterase, and auxiliary activities (**Figure 3A**), respectively. However, proteins containing a glycosyltransferase module, which mediates the formation of glycosidic bonds, were not identified in the induced exoproteome, and of the CAZymes in the induced exoproteome, almost all (112 out of 126) were typical secreted forms (i.e., harboring a signal peptide), 28 were SCRPs, and 49 were homologous to known pathogenic factors in the PHI database, of which 16 have previously been reported to influence the virulence of other microbial pathogens according to the PHI database annotation (**Figure 3B**).

On the basis of abundance variations of proteins identified in the induced exoproteome, CAZyme regulation patterns differed significantly. For example, abundance of almost all CAZymes was significantly up-regulated in the induced exoproteome, with the exception of one protein containing the CBM50 domain which was reduced in abundance (VEDA_09538) (**Figure 3C**; Supplementary Table S6). Results show that 28 of the 30 identified proteins with a greater than ninefold enhanced abundance were CAZymes (**Figure 3C**); the functional clustering of these proteins suggests that they are mainly involved in plant cell wall degradation as 30, 22, and 22 are related to pectin, cellulose, and hemicellulose degradation, respectively (**Figure 3D**). In addition, CAZymes abundance associated with plant cell wall degradation was significantly greater than that of the other identified proteins (**Figure 3E**). Those involved in the degradation of pectin and cellulose were considerably up-regulated, more than 75% at least five times in the CCD medium (**Figure 3E**). These results strongly suggest that enhanced plant cell wall degradation activity plays an important role in the CCD medium because of the presence of cotton cell wall components, and that this mechanism promotes the germination of the *V. dahliae* pathogen and its proliferation in xylems.

### The Induced Exoproteome Is Involved Mainly in Pectin and Cellulose Degradation

To overcome the plant cell wall barrier, phytopathogenic fungi produce enzymes that degrade polymers, particularly pectin and cellulose. Results show that of the CAZymes related to pectin degradation, 30 were up-regulated in the CCD medium, including 13 CAZymes module types (e.g., PL1, PL3, and GH28) (Supplementary Figure S2). Similarly, 22 identified proteins, including nine CAZymes module types (e.g., GH3, GH5, GH7, and CBM1) involved in cellulose degradation were also up-regulated in the CCD medium (Supplementary Figure S2B). Interestingly, abundances of CAZymes involved in pectin and cellulose degradation were also up-regulated in the CCD medium; proteins 26 and 18, respectively, were up-regulated by more than fivefold (Supplementary Figure S2). These results strongly suggest that CAZymes associated with the degradation of pectin and cellulose play a crucial role in *V. dahliae* germination and proliferation in the CCD medium.

**FIGURE 4:**
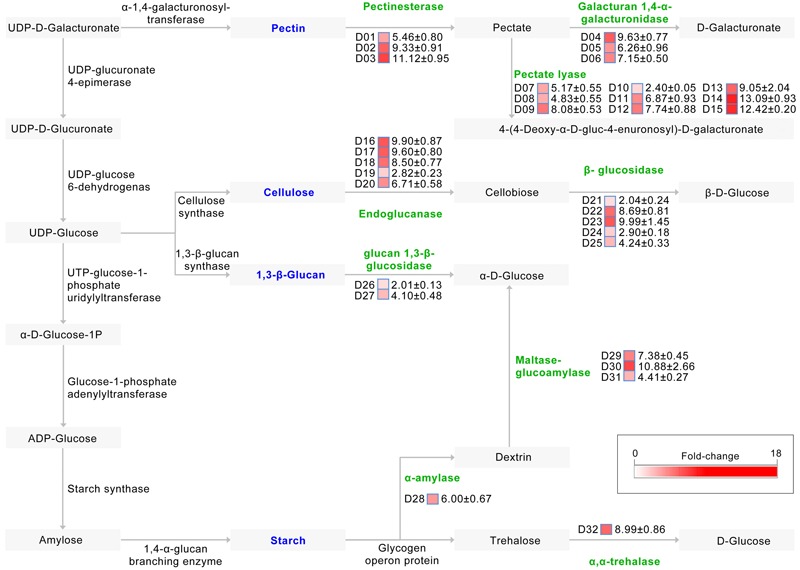
**Induced exproteome involved in plant cell wall degradation.** Identified proteins were matched to a reference pathway using the KEGG database, while the plant cell wall degradation pathway was drawn according to the starch and sucrose metabolism pathways (ko:00500). D01 to D32 represents 32 proteins involve in plant cell wall degradation, the fold change (original value) of identified proteins were presented by the color blocks from white (zero fold-change) to red (the maximum fold-change).

Pathway annotation shows that at least 32 up-regulated CAZymes are involved in starch and sucrose metabolism (ko:00500)^2^, required for the degradation of plant cell walls (**Figure 4**). Indeed, of the proteins involved in pectin degradation, the abundance of 15 which mediate the degradation of pectin to pectate and onward to D-galacturonate or 4-(4-deoxy-α-D-gluc-4-enuronosyl)-D-galacturonate were strongly up-regulated in the CCD medium (**Figure 4**). Similarly, the abundance of 10 proteins, most of which are involved in the degradation of cellulose to cellobiose and then onward to β-D-glucose, were also significantly up-regulated in the CCD medium (**Figure 4**), as were the abundances of proteins involved in the metabolism of other plant cell wall components (e.g., 1,3-β-glucan, and starch) (**Figure 4**). Thus, the *V. dahliae* pathogen recruits CAZymes to participate in plant cell wall degradation (cotton tissue) by breaking down pectin and cellulose.

### The Role of Identified Proteins in *V. dahliae* Virulence

**FIGURE 5:**
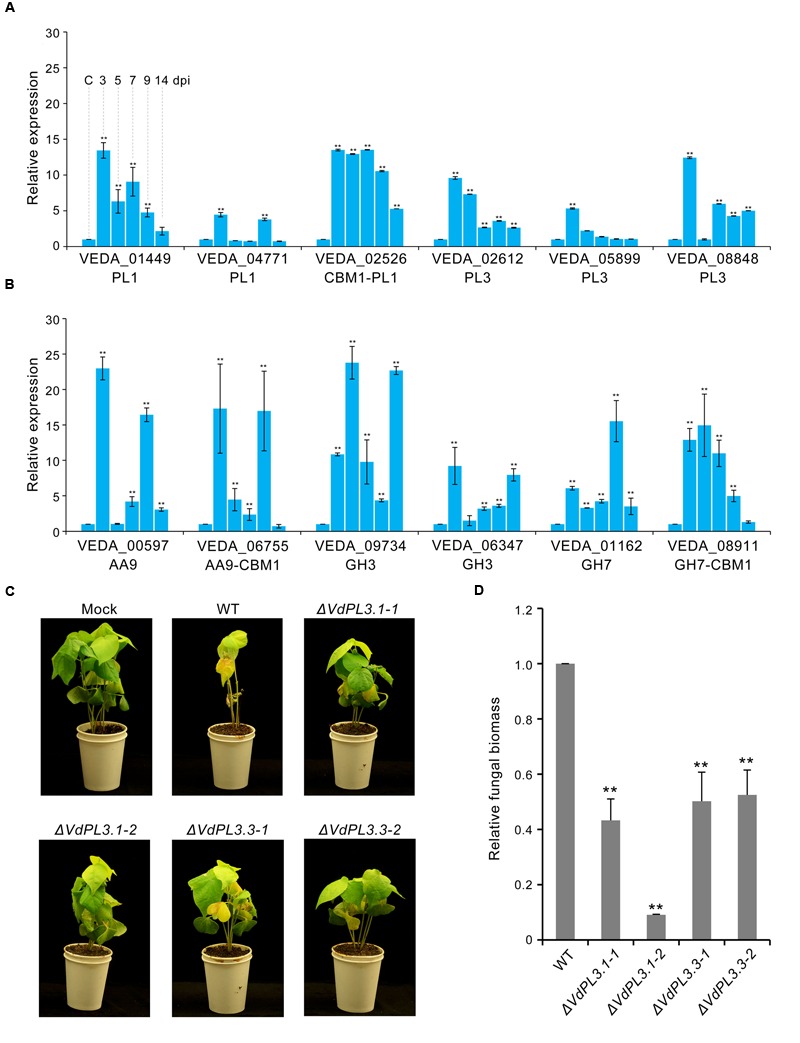
**Virulence function analysis of identified proteins in the exoproteome. (A)** Expression of genes involved in pectin degradation in *V. dahliae* infection on cotton roots determined via qRT-PCR. The control (capital letter C) shows the expression level of genes in a mixture of non-inoculated conidial and cotton root tissue; the housekeeping gene *β-tubulin* (VDAG_10074) was used as an endogenous control. Error bars show standard error, ^∗∗^ denotes statistical significance (*P* ≤ 0.01) of different time points compared to control using an unpaired Student’s *t*-test. **(B)** Expression of genes involved in cellulose degradation by *V. dahliae*. **(C)** Phenotypes of cotton seedlings inoculated with *VdPL3.1* and *VdPL3.3* gene-deletion strains. Two-week-old seedlings of susceptible cotton, *Gossypium hirsutum* L., ‘Junmian No.1,’ were inoculated with sterile water (Mock), wild-type (WT) *V. dahliae*, and the two independent *VdPL3.1* and *VdPL3.3* gene-deletion strains. **(D)** Fungal biomass of the gene-deletion strain on cotton was determined using qRT-PCR. Error bars represent standard error, and ^∗∗^ denotes statistical significance (*P* ≤ 0.05) of gene-deletion strains compared to WT using an unpaired Student’s *t*-test.

To evaluate the pathogenic function of PCWDEs in *V. dahliae*, expression levels of several involved in pectin and cellulose degradation were analyzed. Results show that transcription levels of PCWDEs involved in pectin and cellulose degradation are markedly increased during *V. dahliae* infection of susceptible cotton plants (**Figures 5A,B**), while real-time PCR showed that these levels increased markedly around three dpi, and that the expression of many remained high at 5–7 dpi (**Figures 5A,B**). These periods are generally considered to be the key initial germination and proliferation stages of cotton infection.

In order to further verify the role of PCWDEs in *V. dahliae* pathogenesis, the PL3 subfamily, involved in pectin degradation, was subjected to further analysis. This subfamily exhibits pectate lyase activity, cleaving poly-1, 4-α-D-galacturonan (pectate, the backbone of pectin) to 4-(4-deoxy-α-D-gluc-4-enuronosyl)-D-galacturonate at the non-reducing end (**Figure 4**). The results of this study show that the abundances of three PL3 genes (i.e., VEDA_02612, VEDA_05899, and VEDA_08848, denoted *VdPL3.1*–*VdPL3.3*) were significantly up-regulated 6.87 ± 0.93, 13.09 ± 0.93, and 12.42 ± 0.20 times, respectively, in the CCD medium (Supplementary Figure S2A). These results suggest that PL3 genes thus play an important role in the infection of cotton.

In order to confirm the pathogenic functions of PCWDEs in the induced exoproteome, targeted replacement of the three PL3 genes (i.e., *VdPL3.1*–*VdPL3.3*) by a hygromycin resistance cassette through homologous recombination was pursued, and gene deletion was verified by PCR (Supplementary Figure S3), of which two were used for further analysis in this study. Pathogenicity analysis of these three strains showed that the *ΔVdPL3.1* and *ΔVdPL3.3* mutants were significantly decreased in virulence, leading to a reduction in stunting and no obvious disease symptoms in susceptible cotton compared with the wild-type strain (**Figure 5C**). Unexpectedly, although the transcription level of another PL3 gene, *VdPL3.2*, was also significantly induced during cotton infection, the deletion strain, *ΔVdPL3.2*, exhibited virulence similar to that seen in the wild-type strain (Supplementary Figure S4). This result is likely due to the fact that *V. dahliae* encodes other CAZymes that can complement the pectate lyase function of *VdPL3.2*. Similarly, only a double mutant in *PL3* genes encoding pectate lyase A and D (i.e., *pelA* and *pelD*) in *Nectria hematococca* led to drastically reduced pathogenicity to *Pisum sativum* (Rogers et al., 2000). We conclude that CAZymes in the induced exoproteome of *V. dahliae*, including the pectin lyases VdPL3.1 and VdPL3.3, are involved in plant cell wall degradation and play a crucial role in the pathogenesis of this fungus.

## Discussion

The pathogen *V. dahliae* is the causal agent of plant *Verticillium* wilt disease, causing billions of dollars in annual crop losses (Fradin and Thomma, 2006; Klosterman et al., 2009). Previous studies have shown that the exoproteome, including toxins, effectors, and PCWDEs, likely plays a critical role in *V. dahliae* pathogenesis (Fradin and Thomma, 2006). To date, several secreted proteins have been identified using traditional separation and purification techniques and have been demonstrated to be associated with disease symptoms in susceptible host plants (Buchner et al., 1982, 1989; Nachmias et al., 1985; Meyer et al., 1994; Mansoori et al., 1995; Davis et al., 1998). However, pathogenic factors in the *V. dahliae* exoproteome have so far not been reported. In this study, we show that the *V. dahliae* exoproteome from an CCD medium culture caused more serious wilting and necrosis in cotton cotyledon leaves compared with that from an original CD medium utilizing sucrose as a carbon source. In addition, results show that the impaired cotton phenotype was similar to that of leaves infected by *V. dahliae* inoculation (**Figure 1**), suggesting production of several virulence factors in the CCD medium. Thus, identification of the components of the induced exoproteome using iTRAQ combined with LC-MS/MS could facilitate determination of the mechanisms underlying infection of cotton by *V. dahliae*.

Although several previous studies have reported that crude extracts of the *V. dahliae* exoproteome exhibit virulence to host plants (Buchner et al., 1982; Mansoori et al., 1995; Yuan et al., 2006), active components remain unknown. In this study, 325 proteins showed significant changes in abundance in the CCD medium compared with those in the original CD utilizing sucrose as a carbon source (**Table 1**; Supplementary Table S2). Of these, 69% were secreted and 63% were typical secreted proteins containing a signal peptide (**Table 1**), suggesting protein secretion by *V. dahliae* occurs mainly via the Golgi/ER system. In addition, because 31% of these proteins lacked a signal peptide and were not classified as NCSPs (independent of the Golgi/ER), this suggests that *V. dahliae* may possess as yet unknown secretory mechanisms.

Secreted effectors facilitate colonization of the host plant by pathogens as they modulate host biochemistry and physiology (Stergiopoulos and de Wit, 2009). Previous research has shown that several kinds of effectors are conserved among phytopathogens, including LysM, NLPs, and Ave1 (de Jonge et al., 2012, 2013; Zhou et al., 2012; Santhanam et al., 2013), while others have been described simply as SCRPs with no known function. Our results show that the abundances of 28 SCRPs were significantly up-regulated in the induced exoproteome (**Figure 3B**), suggesting that they likely play an important role in *V. dahliae* pathogenesis. In addition, abundances of two NLP proteins, NLP1 and NLP2 (Zhou et al., 2012; Santhanam et al., 2013), were also significantly enhanced (9.05 ± 2.04 and 3.75 ± 0.17 times, respectively) in the induced exoproteome (Supplementary Table S5), while one LysM effector (VEDA_00253) was also up-regulated 5.16 ± 0.50 times following culture in the CCD medium. These results suggest that secreted effectors are important adaptions of *V. dahliae* to the cotton vascular system environment, likely protecting this pathogen from the deleterious effects of host defenses.

A few previous studies have reported that hydrolases are important to the pathogenicity of *V. dahliae* (Puhalla and Bell, 1981; Pegg and Brady, 2002). For example, hydrolases involved in carbohydrate metabolism were shown to be affected following *VdSNF1* deletion, leading to failed colonization and the induction of disease symptoms on tomato and eggplant (Tzima et al., 2011). In this study, the functional clustering of up-regulated secretory proteins showed that the number of hydrolases significantly increased in the CCD medium (**Figure 2**). Previous transcriptome analyses have also demonstrated increased hydrolase expression during the interaction between *V. dahliae* and its host plant (Xiong et al., 2014, 2015), and this has also been reported in other phytopathogens (Lysøe et al., 2011; Mathioni et al., 2011; O’Connell et al., 2012). Carbohydrate enzymes are necessary for the growth of pathogenic fungi in the presence of low carbohydrate levels, as well as possibly also for the penetration of plant roots by soil-borne fungi gaining access to the xylem (Kubicek et al., 2014). One previous comparative genomics study reported that the *V. dahliae* genome encodes a greater number of CAZymes than is the case in other fungi, suggesting an enhanced capacity to degrade plant cell walls (Klosterman et al., 2011). Indeed, analysis of the secretome of the closely related vascular wilt pathogen *V. alboatrum* in a simulated xylem fluid showed that carbohydrate hydrolases were more abundant in lethal strains than in others (Mandelc and Javornik, 2015). Thus, as shown here, to adapt to the conditions of the CCD medium, *V. dahliae* produced numerous extracellular proteins that participate in carbohydrate metabolism, most of which are significantly up-regulated in the presence of low nutrient levels (**Figure 3**). Our results confirm that carbohydrate hydrolases play a crucial role in the virulence of vascular phytopathogens by facilitating their evasion of host defenses. This is necessary for the successful growth of phytopathogens in carbohydrate-poor environments, facilitates fungal colonization, and the production of survival structures in plant tissue.

The main cell wall components in dicotyledonous plants include pectic polysaccharides, cellulose microfibrils, hemicelluloses, and glycoproteins (Carpita and Gibeaut, 1993). This primary cell wall functions as a barrier to prevent the penetration of pathogens and infection (Hématy et al., 2009). To overcome this barrier, pathogens produce PCWDEs that degrade polysaccharides and obtain nutrients (King et al., 2011). Genomic sequencing has revealed that the pectate lyase (i.e., PL1, PL3, and PL9), rhamnogalacturonan lyase (i.e., PL4 and PL11), and CBM1 families of enzymes, which mediate the cleavage of pectin and cellulose during infections, are increased in abundance in *V. dahliae* (Klosterman et al., 2011), associated with enhanced plant cell wall degradation. In this study, analysis of the expression of PCWDE genes *in planta* demonstrates their involvement in *V. dahliae* pathogenicity (**Figure 5A**); highest expression occurred at 3 dpi, when the fungus entered the root xylem vessels and proliferated into the vascular system (Gold and Robb, 1995; Heinz et al., 1998; Chen et al., 2004).

Several pectinolytic enzymes (i.e., polygalacturonase, pectate lyase, and pectinesterase) are known to be capable of necrotizing plant tissues *in vitro* and causing wilt symptoms *in vivo* (Mussel and Strause, 1972; Cooper and Wood, 1980; Huang and Mahoney, 1999). Thus, to confirm the pathogenicity of these pectin-lytic enzymes, the functions of three pectate lyase genes were evaluated using a genetic knockout experiment. However, although the VdLs.17 strain encodes for 11 *PL3* genes all of which potentially encode secreted proteins likely involved in pectin degradation (Klosterman et al., 2011), few previous studies have evaluated the role of these genes in *V. dahliae* pathogenicity. Results presented here show that these three *PL3* genes were markedly up-regulated in the induced exoproteome as well as *in planta* after *V. dahliae* infection (**Figure 5A**). Indeed, the virulence to cotton plants of strains containing the *VdPL3.1* and *VdPL3.3* deletions were markedly reduced (**Figures 5C,D**). Our findings suggest that PCWDEs function as important virulence factors in *V. dahliae* pathogenesis.

## Conclusion

The *V. dahliae* exoproteome purified using the CCD medium caused serious wilting and necrosis in cotton cotyledons. A number of potential pathogenicity-related factors were identified in the exoproteome via iTRAQ combined with LC-MS/MS. Protein abundance data suggest that pathogenicity-related factors in the exoproteome are significantly up-regulated, particularly CAZymes involved in carbohydrate metabolism. Further analysis indicates a marked increase in PCWDE levels in the exoproteome, which participate in pectin and cellulose degradation. Indeed, PL3 deletion mutants exhibited reduced virulence against cotton, which suggests that some PCDWEs in the induced exoproteome of *V. dahliae* function as virulence factors. Our findings strongly suggest that hydrolytic enzymes in the induced exoproteome of *V. dahliae* are closely related to plant cell wall degradation, and thus likely play a crucial role in both host colonization and proliferation in the unique plant vascular system environment.

## Author Contributions

XD conceived the study and designed all experiments, while JC performed the data analysis and interpretation, HX performed the pathogenicity characterization experiment and wrote the manuscript, YG and DZ performed the target gene deletion, and LL and YB performed the gene expression analysis. All authors have read, commented on, and approved the manuscript.

## Conflict of Interest Statement

The authors declare that the research was conducted in the absence of any commercial or financial relationships that could be construed as a potential conflict of interest.

## Footnotes

1 http://www.phi-base.org/

2 http://www.genome.jp/kegg/pathway.html
