# Supplementary material for: Characterization of the Verticillium dahliae Exoproteome Involves in Pathogenicity from Cotton-Containing Medium
Source: Front Microbiol. 2016 Oct 28;7:1709. doi: 10.3389/fmicb.2016.01709 (PMC5083787; doi:10.3389/fmicb.2016.01709)
Supplement: Supplementary file 8 [file Image_1.PDF]

Supplementary Material

Functional analysis of the *Verticillium dahliae* exoproteome in a simulated cotton xylem environment

Jieyin Chen[#], Hongli Xiao[#], Yuejing Gui, Dandan Zhang, Lei Li, Yuming Bao, Xiaofeng Dai[*]

[#] These authors contributed equally to this work

*** Correspondence:** Xiao-Feng Dai: daixiaofeng_caas@126.com

1 Supplementary Figures

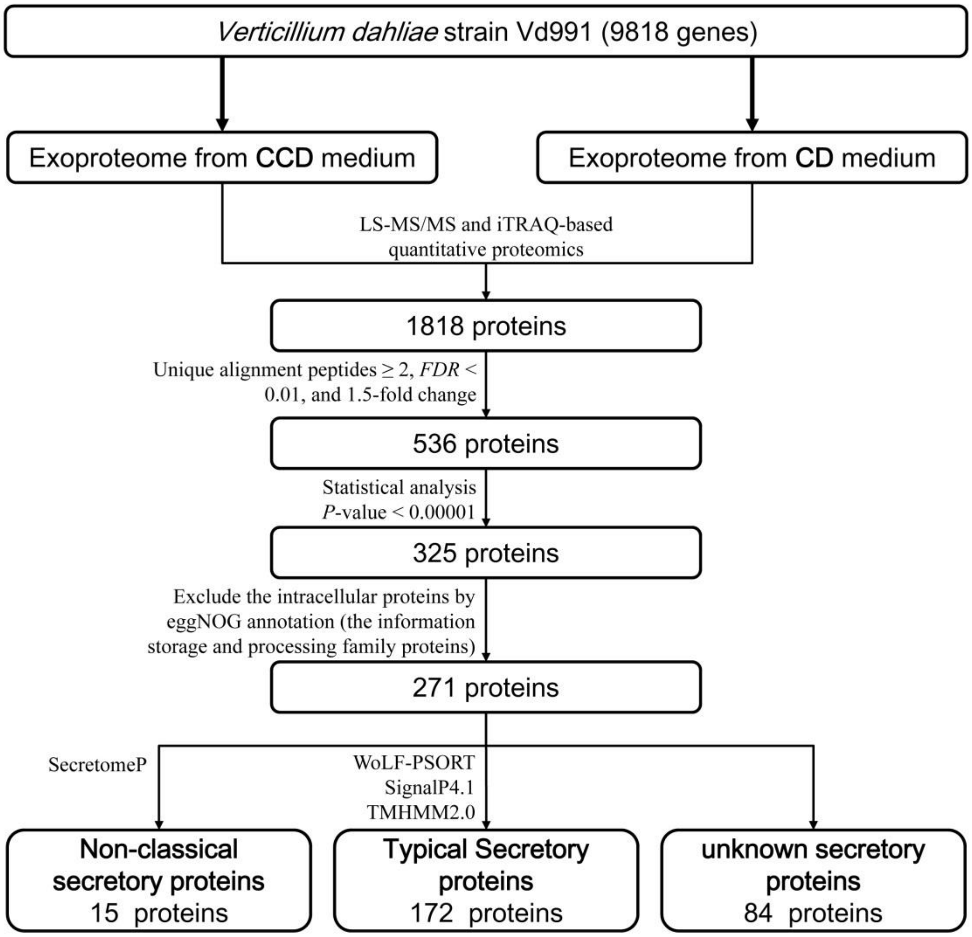

Figure S1. The experiment flow of identify the induce exproteome in cotton-containing medium. CCD medium, cotton-containing C'zapek-Dox medium; CD, C'zapek-Dox medium.

A

Gene-ID	Sub-family	Fold-change
VEDA_01449 (D07)	PL1	5.17±0.55
VEDA_02470 (D08)	PL1	4.83±0.55
VEDA_02509 (D09)	PL1	8.08±0.53
VEDA_02526 (D10)	CBM1, PL1	2.40±0.05
VEDA_04771 (D12)	PL1	7.74±0.88
VEDA_05049 (D13)	PL1	9.05±2.04
VEDA_05925	PL1	1.74±0.18
VEDA_02612 (D11)	PL3	6.87±0.93
VEDA_05899 (D14)	PL3	13.09±0.93
VEDA_08848 (D15)	PL3	12.42±0.20
VEDA_07870	PL4	1.56±0.14
VEDA_09382	PL4	7.87±0.57
VEDA_02653	PL9	12.29±1.59
VEDA_01417 (D04)	GH28	9.63±0.77
VEDA_02503 (D05)	GH28	6.26±0.96
VEDA_03250	GH28	9.11±1.27
VEDA_05861	GH28	6.79±0.64
VEDA_08583 (D06)	GH28	7.15±0.50
VEDA_01470	GH35	5.89±0.40
VEDA_07914	GH51	5.59±0.45
VEDA_01287	GH78	7.65±0.52
VEDA_07694	GH78	7.13±1.30
VEDA_06283	GH93	8.75±0.71
VEDA_06060	GH105	8.46±1.57
VEDA_03251 (D01)	CE8	5.46±0.80
VEDA_05701 (D02)	CE8	9.33±0.91
VEDA_09412 (D03)	CE8	11.12±0.95
VEDA_03163	CE12	7.56±2.12
VEDA_05702	CE12	7.42±0.76
VEDA_07932	CE12	11.70±0.49

B

Gene-ID	Sub-family	Fold-change
VEDA_00597	AA9	17.39±1.16
VEDA_03687	AA9	6.64±0.86
VEDA_06054 (D20)	AA9, CBM1	6.71±0.58
VEDA_06755	AA9, CBM1	8.54±0.41
VEDA_00540 (D21)	GH3	2.04±0.24
VEDA_03908 (D22)	GH3	8.69±0.81
VEDA_06347 (D23)	GH3	9.99±1.45
VEDA_07143 (D24)	GH3	2.90±0.18
VEDA_09734 (D25)	GH3	4.24±0.33
VEDA_04966	GH5	8.54±0.66
VEDA_05572	GH5	7.29±0.14
VEDA_07806	GH5	12.04±1.61
VEDA_05881	GH5, CBM1	11.68±0.54
VEDA_03168 (D18)	CBM1, GH5	8.50±0.77
VEDA_00136 (D16)	CBM1, GH5, CBM46	9.90±0.87
VEDA_08813	GH6	6.00±0.56
VEDA_05953	CBM1, GH6	14.09±1.29
VEDA_01162 (D17)	GH7	9.60±0.80
VEDA_01436	GH7, CBM1	12.50±1.93
VEDA_08911	GH7, CBM1	11.99±2.85
VEDA_00253	GH45	5.16±0.50
VEDA_04614 (D19)	CBM63	2.82±0.23

0 Fold-change 18

Figure S2. Information of the enhanced abundance proteins associated to pectin and cellulose degradation. The bracket containing “Dx” number represents the gene matched to the starch and sucrose metabolism pathway by BLAST analysis. The fold change is the original value. (A) Proteins involved in pectin degradation. (B) Proteins involved in cellulose degradation.

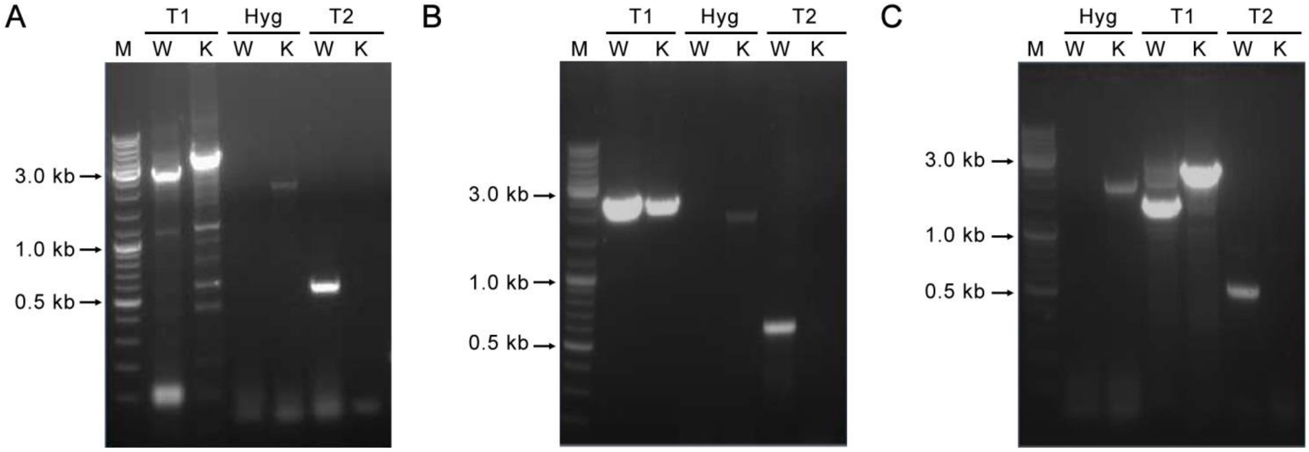

Figure S3. Evidences for target gene deletion of three *PL3* genes. W, wild type Vd991; K, knock-out mutant; M, DNA Marker. Hyg, hygromycin phosphotransferase gene fragment, only presents in the knock out mutant; T1, test by the primers in the flanking sequence of target genes, the size of amplicons are different due to different length between hygromycin phosphotransferase gene and target gene; T2, test by the primers within target genes, only present in the wild type. (A) *VdPL3.1*. (B) *VdPL3.2*. (C) *VdPL3.3*.

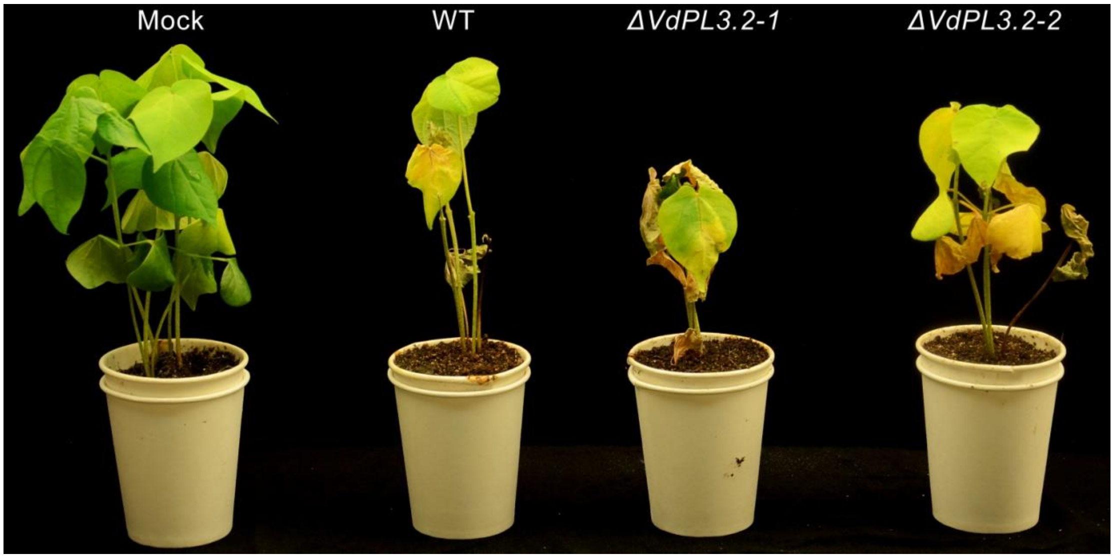

Figure S4. Phenotypes of cotton seedlings inoculated with the *VdPL3.2* gene-deletion strains. Two-week-old seedlings of susceptible cotton of *Gossypium hirsutum* cv. Junmian1 were inoculated with sterile water (Mock), wild-type *V. dahliae* (WT), the *VdPL3.2* gene-deletion strains. The disease symptom phenotype was photographed three weeks after inoculation.
